# Comparative Proteomic Analysis of Nodulated and Non-Nodulated *Casuarina glauca* Sieb. ex Spreng. Grown under Salinity Conditions Using Sequential Window Acquisition of All Theoretical Mass Spectra (SWATH-MS)

**DOI:** 10.3390/ijms21010078

**Published:** 2019-12-20

**Authors:** Inês Graça, Vera M. Mendes, Isabel Marques, Nuno Duro, Mário da Costa, José C. Ramalho, Katharina Pawlowski, Bruno Manadas, Cândido P. Pinto Ricardo, Ana I. Ribeiro-Barros

**Affiliations:** 1 PlantStress&Biodiversity Lab, Linking Landscape, Environment, Agriculture and Food (LEAF), Instituto Superior de Agronomia (ISA), Universidade de Lisboa, Tapada da Ajuda, 1349-017 Lisbon, Portugal; inesgraca@hotmail.com (I.G.); isabelmarques@isa.ulisboa.pt (I.M.); nuno_m_h_d@hotmail.com (N.D.); mjadacosta@gmail.com (M.d.C.); cochichor@mail.telepac.pt (J.C.R.); 2 Plant Biochemistry Lab, Instituto de Tecnologia Química e Biológica, Universidade NOVA de Lisboa, Av. República, Quinta do Marquês, 2780-157 Oeiras, Portugal; ricardo@itqb.unl.pt; 3 CNC—Center for Neuroscience and Cell Biology, Universidade de Coimbra, UC Biotech—Parque Tecnológico de Cantanhede, Núcleo 04, Lote 8, 3060-197 Cantanhede, Portugal; vera3m@gmail.com (V.M.M.); bmanadas@gmail.com (B.M.); 4 GeoBioTec, Faculdade de Ciências e Tecnologia (FCT), Universidade NOVA de Lisboa (UNL), 2829-516 Caparica, Portugal; 5 Department of Ecology, Environment and Plant Sciences, Stockholm University, 106 91 Stockholm, Sweden; katharina.pawlowski@su.se

**Keywords:** actinorhizal plants, *Casuarina glauca*, *Frankia*, proteomics, salt stress

## Abstract

*Casuarina glauca* displays high levels of salt tolerance, but very little is known about how this tree adapts to saline conditions. To understand the molecular basis of *C. glauca* response to salt stress, we have analyzed the proteome from branchlets of plants nodulated by nitrogen-fixing *Frankia* Thr bacteria (NOD^+^) and non-nodulated plants supplied with KNO_3_ (KNO_3_^+^), exposed to 0, 200, 400, and 600 mM NaCl. Proteins were identified by Short Gel, Long Gradient Liquid Chromatography coupled to Tandem Mass Spectrometry and quantified by Sequential Window Acquisition of All Theoretical Mass Spectra -Mass Spectrometry. 600 proteins were identified and 357 quantified. Differentially Expressed Proteins (DEPs) were multifunctional and mainly involved in Carbohydrate Metabolism, Cellular Processes, and Environmental Information Processing. The number of DEPs increased gradually with stress severity: (i) from 7 (200 mM NaCl) to 40 (600 mM NaCl) in KNO_3_^+^; and (ii) from 6 (200 mM NaCl) to 23 (600 mM NaCl) in NOD^+^. Protein–protein interaction analysis identified different interacting proteins involved in general metabolic pathways as well as in the biosynthesis of secondary metabolites with different response networks related to salt stress. Salt tolerance in *C. glauca* is related to a moderate impact on the photosynthetic machinery (one of the first and most important stress targets) as well as to an enhancement of the antioxidant status that maintains cellular homeostasis.

## 1. Introduction

Actinorhizal plants are a group of perennial dicots from eight different plant families that establish root-nodule symbiosis with N_2_-fixing soil actinobacteria of the genus *Frankia*. These plants are highly resilient to extreme environments [1], constituting an important model to study stress adaptation and biological nitrogen fixation. However, broad, interdisciplinary and integrate research to ascertain the contribution of symbiotic *Frankia* to the environmental plasticity of actinorhizal species has emerged only recently, mostly focusing on salt stress in Casuarinaceae, particularly in the model species *Casuarina glauca* Sieb. ex Spreng e.g., Santos et al., Ribeiro et al., Batista-Santos et al., Mansour et al., Ribeiro-Barros et al., Jorge et al., and Ngom et al. [2,3,4,5,6,7,8,9]. *C. glauca* is a tropical tree widely used to prevent desertification and erosion, as well as to rehabilitate poor and degraded soils [1,10]. Such capacity does not necessarily depend on the symbiosis with *Frankia*, although some strains may enhance stress tolerance [4,5,6,9,11,12,13].

This aspect is particularly relevant in the context of the prospective environmental challenges, namely extended drought periods, raising temperatures and soil salinization [14,15]. The latter is a crucial limiting factor in agro-forestry and is predicted to increase, considering extreme weather events and rising seawater levels [16,17]. Indeed, by the middle of this century salinity is expected to affect up to 50% of all cultivable land [18]. Additionally, spreading agriculture to arid and semi-arid regions will demand intensive irrigation schemes, promoting secondary soil salinization due to an imbalance between water input (irrigation or rainfall) and water use (transpiration) [15].

Plant adaptation or tolerance to salinity stress results from a complex network of complementary molecular, cellular, metabolic, and physiological events [19,20]. Recently, the mechanisms underlying salt stress tolerance in *C. glauca* and the contribution of symbiotic nitrogen fixation have been thoroughly analysed by our team. For that, we have examined the impact of increasing NaCl concentrations (200, 400 and 600 mM) on plant growth, mineral contents, water relations, photosynthesis-related parameters and non-structural sugars [4,12,13], membrane integrity and the control of oxidative stress [21], as well as the metabolome of nodules, roots and branchlets from nodulated and non-nodulated *C. glauca* plants [7,8,22]. According to Batista-Santos et al. [4], the *C. glauca* ecotype used in these studies exhibits outstanding salt stress tolerance showing the first stress symptoms, i.e., phenotypic changes (chlorosis and necrosis of branchlets, reduction of stem diameter and biomass decrease in both KNO_3_^+^ and NOD^+^ groups; and swollen nodules with salt crystals at the surface in NOD^+^ plants), and the impairment of photochemical (e.g., photosynthetic electron flow) and biochemical (e.g., activity of photosynthetic enzymes) parameters only at 600 mM NaCl. This has been associated with a remarkably low level of tissue dehydration combined with strong osmotic adjustments, features that are essential to maintain a potential gradient of water influx and to sustain metabolic activity [15]. In fact, analysis of the primary metabolome of *C. glauca* branchlets [7,8] indicated that modifications in the pattern of accumulation of osmoprotectant molecules (neutral sugars, proline, and ornithine) were involved in salt stress tolerance. On the other hand, despite the reduction of net photosynthesis at salt concentrations of 400 mM NaCl and above, the photosynthetic assimilation potential was mainly kept at extraordinary high salt levels, indicating down-regulation of photosynthesis rather than severe damages [4]. This tolerance is likely related to the control of the production of reactive oxygen species (ROS) in plant cells [22,23,24,25]. Indeed, photosynthetic branchlets of *C. glauca* showed a marked increase of the activity of several anti-oxidative enzymes (e.g., superoxide dismutase, ascorbate peroxidase, glutathione reductase and catalase), helping to preserve membrane stability up to 400 mM NaCl [21]. Notably, this tolerance seems to be largely independent of the presence of an active N_2_-fixing symbiosis, as revealed by the fact that at 200 mM NaCl the *Frankia* strain used (Thr) was no longer able to fix nitrogen in *C. glauca* nodules [12]. However, a mutualistic response to salt stress is probably dependent on the genotype combination of macro- and microsymbiont. In fact, preliminary growth tests of free-living *Frankia* Thr in the presence of increasing NaCl concentrations indicated that this strain was indeed salt tolerant, maintaining a normal growth curve at least up to 600 mM NaCl (unpublished data). Also, studies developed by other research groups revealed that despite the fact that salt stress response is rather diverse among *Frankia* strains in vitro [26], a similar impact of salt stress was observed in *C. glauca* plants nodulated by “salt tolerant” and “salt sensitive” strains, respectively [9]. In short, the level of salt tolerance *in vitro* did not predict the level of salt tolerance *in planta.* Nevertheless, it is also noteworthy that the tolerance limits of the *C. glauca* ecotype used by Ngom et al. [9] were lower than the limits of the *C. glauca* ecotype used in our studies [4,12,21], i.e., 200 mM vs. 600 mM NaCl.

Systems biology approaches are the best way to thoroughly dissect the complex interactions within biological systems, and the pathways involved in stress adaptation, as well as to deepen our understanding of the expression patterns and functions of the response-associated genes and proteins [20,27]. For the investigation of the roles that proteins play in cell metabolism, the use of proteomics is essential. This approach provides the elements required for the systematic analysis of protein properties—cellular levels, post-translational modifications, and interactions—in tissues exposed to an array of different environmental conditions [28]. One of the main bottlenecks for the use of proteomics is the lack of robust bioinformatic tools with algorithmic solutions to process mass spectroscopy (MS) data, which are lagging behind the substantial advances in instrumentation and isolation protocols [29,30]. In the present study, we report the suitability of the Sequential Window Acquisition of All Theoretical Mass Spectra-Mass Spectrometry (SWATH-MS) technique [31] to study salt stress tolerance in plants, using as a model the proteome of *C. glauca*.

SWATH-MS is based on sequential time- and mass-segmented acquisition, which generates fragment ion spectra of all precursors in two user-defined dimensions, retention time and m/z space, resulting in complex fragmentation maps. This is an alternative strategy that combines high specificity data-independent acquisition method with a novel targeted data extraction approach to mine the resulting fragment ion data sets [32]. The interpretation of highly specific multiplexed data sets required the development of fundamentally different data analysis strategy, which uses previously acquired information contained in spectral libraries to mine the fragmentation maps for targeted extraction and quantitation of specific peptides of interest. The accuracy and consistency of SWATH-MS was demonstrated to be comparable to the Selected Reaction Monitoring (SRM) approach [32]. One of the important advantages of the former, alleviating most constrains of present proteomics methods, is the iterative retrospective remining of the acquired data sets for targeted extraction. This approach offers unprecedented possibilities for the qualitative and quantitative profiling not only in proteomics but also in metabolomics and lipidomics. SWATH has high quantification accuracy and precision and can provide very detailed information on low-abundance proteins [32].

As one of the first and most important stress targets is the photosynthetic machinery and photosynthetic efficiency reflects the ability of plants to cope with stress [15,16], and in order to complement previous studies [4,7,8,12,21], at this stage of our research we analysed the impact of increasing salt concentrations (200, 400 and 600 mM NaCl) on the proteome of *C. glauca* branchlets from nodulated plants, relying on symbiotic nitrogen fixation (NOD^+^), and non-nodulated control plants, supplied with nitrate as nitrogen source (KNO_3_^+^).

## 2. Results

### 2.1. Identification and Quantification of Casuarina glauca Proteins in Response to Salinity Stress

To evaluate the proteome changes in branchlets of *Casuarina glauca* plants subjected to increasing salt concentrations, a comparative proteomic analysis was performed. A total of 412, 243, 385 and 376 protein species, respectively, were detected in branchlets of non-nodulated (KNO_3_^+^) plants under control (0 mM NaCl), 200, 400 and 600 mM NaCl conditions, respectively. For nodulated plants (NOD^+^), a total of 216, 135, 219 and 337 protein species were detected at the same salt concentrations.

**Figure 1:**
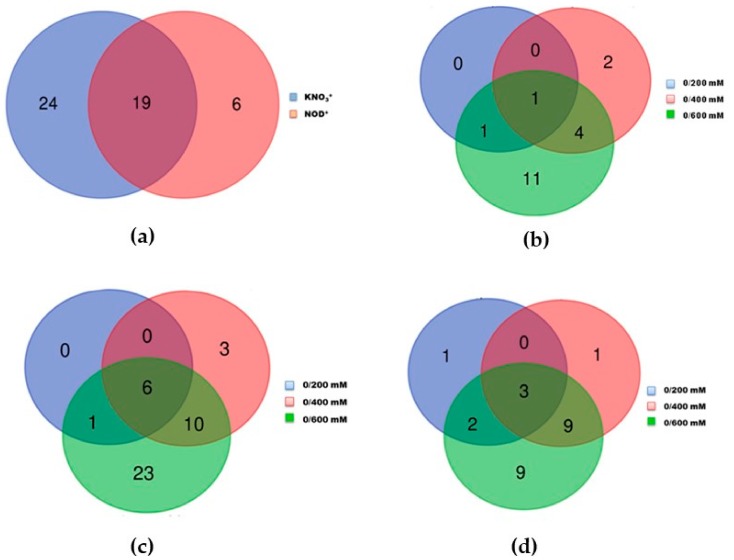
Venn diagrams showing the number of overlapping differentially expressed proteins (DEPs): (**a**) in KNO_3_^+^ vs. NOD^+^ plants; (**b**) between KNO_3_^+^ and NOD^+^ plants at each salt concentrations; (**c**) in control (0 mM NaCl) vs. salt-stressed (200, 400 and 600 mM NaCl) plants for KNO_3_^+^; (**d**) in control (0 mM NaCl) vs. salt-stressed (200, 400 and 600 mM NaCl) plants for NOD^+^ plants.

Using a false discovery rate (FDR) of 5% to gauge the statistical significance of the differences in protein amounts, quantification was possible for a total of 357 protein species. Only the proteins identified with at least 3 different peptides, with a *p*-value below 0.05 and a fold change of at least 1.5 (0.67 > ratio > 1.5) were selected as up- or down-regulated. Based on this cut-off, the number of differentially expressed proteins (DEPs) was 43 in KNO_3_^+^ plants against 25 in NOD^+^ plants, from which 19 were common (Figure 1a). The number of common DEPs within each salt concentration ranged from 2 (out of the 19 DEPs) at 200 mM NaCl to 17 at the highest salt concentration (600 mM NaCl) (Figure 1b).

### 2.2. Differentially Expressed Proteins in Photosynthetic Branchlets of KNO_3_^+^ and NOD^+^ Plants

In both KNO_3_^+^ and NOD^+^ plants, the number of DEPs increased with increasing salt concentrations. For KNO_3_^+^ plants, changes were observed in 7 out of 43 proteins at 200 mM NaCl, 19 proteins at 400 mM NaCl, and 40 proteins at 600 mM NaCl (Figure 1c). For NOD^+^ plants the equivalent values were 6, 13, and 23 out of 25 proteins (Figure 1d). From the set of 43 DEPs in KNO_3_^+^ plants, 3, 13 and 37 were up-regulated, and 4, 6, and 3 down-regulated at 200, 400 and 600 mM NaCl, respectively. As regards the set of 25 DEPs in NOD^+^ plants, 2, 4 and 15 were up-regulated, and 4, 9, and 8 down-regulated at 200, 400 and 600 mM NaCl, respectively. A complete list of the proteins referred in the Venn diagrams is given in Supplementary Tables S1 and S2.

### 2.3. Functional Annotation and Classification of the Identified Proteins

**Figure 2:**
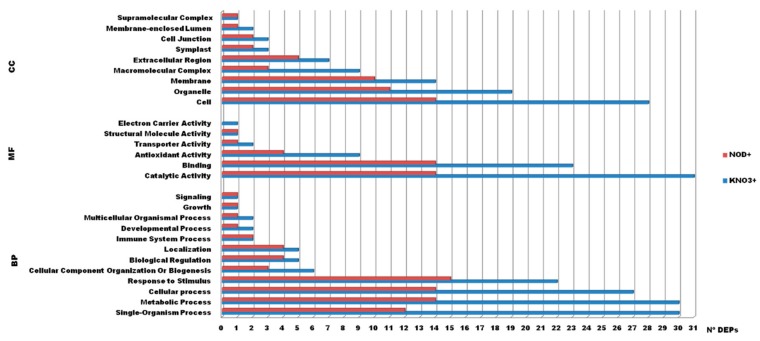
Gene ontology enrichment analysis of the identified differentially expressed proteins (DEPs) from *C. glauca* branchlets at different salt concentrations for KNO_3_^+^ and NOD^+^ plants. The DEPs were grouped into three hierarchically structured terms (y-axis): biological processes (BP), cellular components (CC), and molecular function (MF). The x-axis indicates the number DEPs in specific categories. The blue column represents the 43 DEPs from KNO_3_^+^ and the red column the 25 DEPs from NOD^+^.

To obtain further knowledge on the biological functions of salinity-responsive proteins in *C. glauca* branchlets, all DEP sequences were annotated with three sets of gene ontologies (GOs): Biological Processes (BP), Cellular Components (CC), and Molecular Functions (MF), in 26 and 27 GO terms for KNO_3_^+^ and NOD^+^ plants, respectively (Figure 2). In both plant groups most of the DEPs were multifunctional. In KNO_3_^+^ plants, the major functional BP categories were single-organism and metabolic processes and cellular processes (30 out of 43 DEPs in each category), followed by cellular processes (27) and response to stimulus (22). For MF, catalytic activity (31), binding proteins (23), and antioxidant enzymes (9) were the most abundant groups, whereas cell (28), organelle (19), and membrane (14) proteins accounted for the most abundant CC proteins. In NOD^+^ plants, the major functional BP categories were response to stimulus (15 out of 25), metabolic and cellular processes (14 in each category), and single-organism process proteins (12). Catalytic activity and binding proteins (14 in each category) and antioxidant enzymes (4) were the most abundant MF groups, and cell (14), organelle (11), and membrane proteins (10) were the most represented CC proteins.

**Figure 3:**
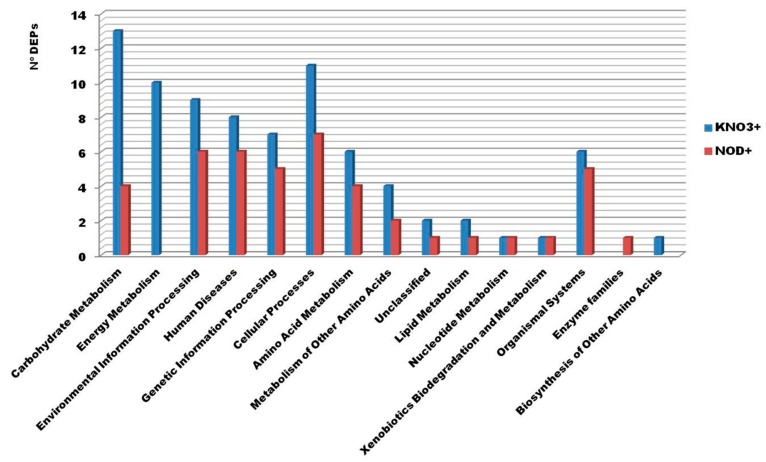
Kyoto Encyclopedia of Genes and Genomes (KEGG) enrichment analysis of the identified differentially expressed proteins (DEPs) from *C. glauca* branchlets at different salt concentrations for KNO_3_^+^ and NOD^+^ plants. The X-axis indicates de KEGG pathway and the Y-axis the number of DEPs assigned to a specific pathway. The blue column represents the 43 DEPs from KNO_3_^+^ and the red column the 25 DEPs from NOD^+^.

Complementarily, Kyoto Encyclopedia of Genes and Genomes (KEGG) analysis was used to understand the biochemical pathways of differentially expressed proteins. KNO_3_^+^ and NOD^+^ DEPs were categorized into 12 and 13 functional categories, respectively (Figure 3). The most represented categories for KNO_3_^+^ and NOD^+^ plants were: Carbohydrate Metabolism (13 out of 43 vs. 4 out of 25), Cellular Processes (11 vs. 7), and Environmental Information Processing (9 vs. 6), respectively. Energy Metabolism (10 DEPs) was also an important category in KNO_3_^+^ plants. Proteins involved in Amino Acid Biosynthesis and Metabolism, accounted for 11 vs. 6 DEPs in KNO_3_^+^ and NOD^+^, respectively, despite the low values of each sub-category. Interestingly, Genetic Information Processing (7 in KNO_3_^+^ vs. 5 NOD^+^) was also among the major categories. Finally, proteins involved in Lipid Metabolism were among the less frequent categories (2 in KNO_3_^+^ vs. 1 in NOD^+^).

### 2.4. Hierarchical Clustering Analysis

**Figure 4:**
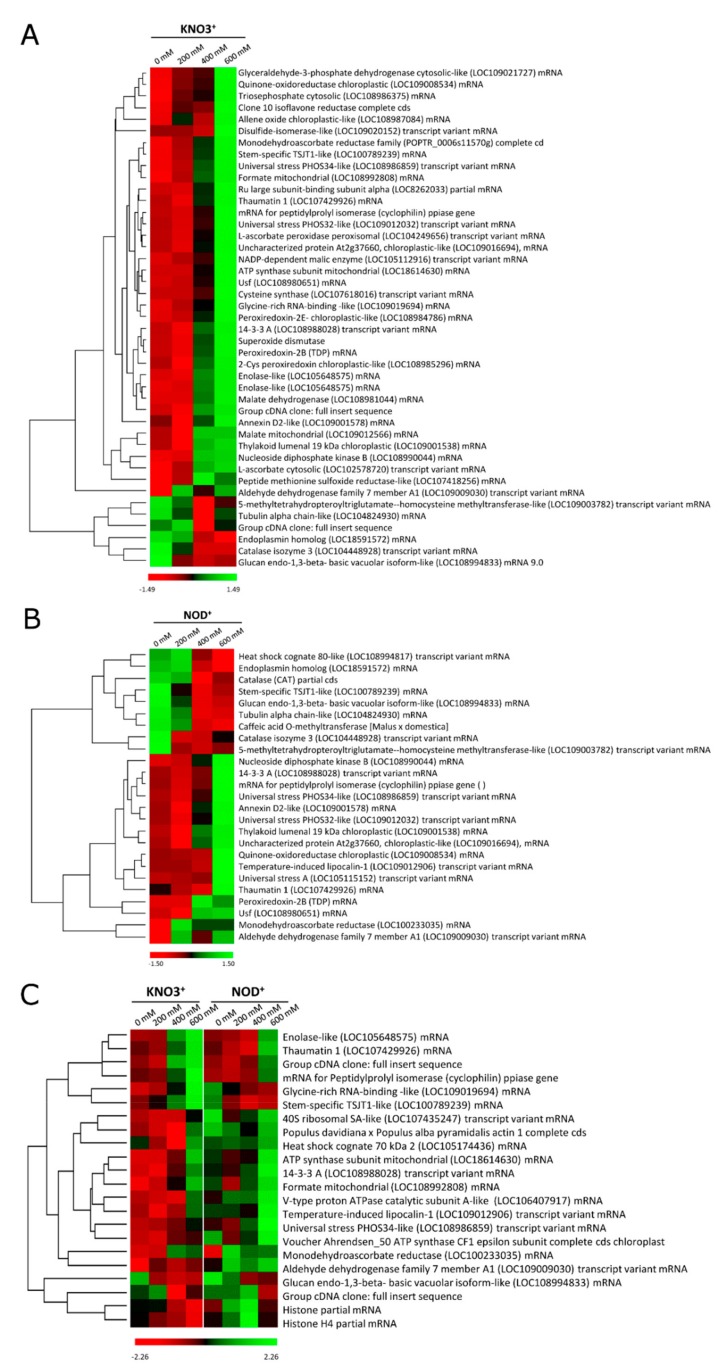
Hierarchical clustering analysis depicting the expression patterns of differentially expressed proteins (DEPs) identified under control (0 mM NaCl), and different salinity conditions (200, 400 and 600 mM NaCl): (**A**) KNO_3_^+^; (**B**) NOD^+^; (**C**) KNO_3_^+^ vs. NOD^+^ plants. Rows represent proteins with *p* < 0.05 and a fold change of at least 1.5 in comparison with the control condition. The proteins that decreased and increased in abundance are indicated in red and green, respectively. The intensity of the colors increases as the expression differences increase, as shown in the bar at the bottom.

Taking into account the statistical parameters considered, a total of 43 (KNO_3_^+^) and 25 (NOD^+^), salt-responsive proteins were grouped by hierarchical clustering. In both plant groups, two main clusters were formed with a marked variation in protein abundance observed from 400 mM NaCl onwards (Figure 4a,b). As indicated above, in both plant groups most DEPs were up-regulated by salt stress. In KNO_3_^+^ plants, these proteins included enzymes involved in (i) ROS defense (e.g., monodehydroascorbate reductase, ascorbate peroxidases, and superoxide dismutase); (ii) glycolysis and TCA cycle (e.g., glyceraldehyde-3-phosphate dehydrogenase, cytosolic NADP-malic enzyme, NAD-dependent malate dehydrogenase); (iii) photosynthetic metabolism (e.g., quinone-oxireductase, thylakoid luminal 19 kDa protein); and (iv) stress-responsive processes (e.g., annexin, allene oxide cyclase, universal-stress protein, thaumatin-like protein, cyclophilin). The salt stress-induced proteins in NOD^+^ plants included a very similar set of categories, that is enzymes involved in (i) ROS defence (e.g., monodehydroascorbate reductase, temperature-induced lipocalin, thioredoxin-dependent peroxidase); (ii) photosynthesis (e.g., quinone-oxireductase, thylakoid luminal 19 kDa); and (iii) stress-responsive proteins (e.g., lipocalin; universal-stress protein, thaumatin). Interestingly, enzymes involved in the respiratory pathway were not among the NOD^+^ DEPs. The variation patterns of KNO_3_^+^ vs. NOD^+^ (Figure 4c and Supplementary Table S3) followed the same trend (up or down), although the protein levels in the latter were generally higher.

### 2.5. Protein-Protein Interaction Network

**Figure 5:**
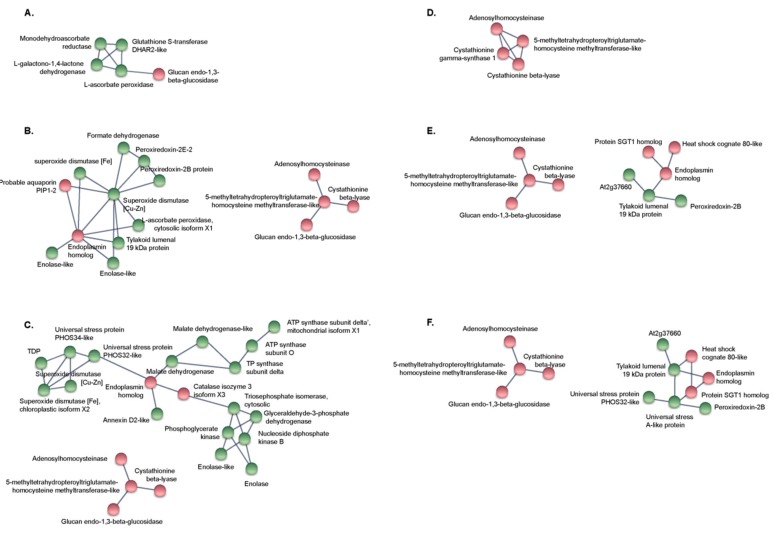
Protein-protein interaction networks of differentially expressed proteins (DEPs) in KNO_3_^+^ (**A**–**C**) and NOD^+^ (**D**–**E**) plants, with different salinity conditions: 200 mM (**A**,**D**), 400 mM (**B**,**E**) and 600 mM (**C**,**F**) NaCl. Green and red nodes represent DEPs with increased and decreased abundance under salt stress, respectively.

Analysis of Protein-Protein Interaction (PPI) networks identified a main group of five interacting proteins involved in general “Metabolic pathways” in KNO_3_^+^ subjected to 200 mM NaCl (Figure 5A). At 400 and 600 mM NaCl, two networks were found. One clustered four proteins involved in “Metabolic pathways” and “Biosynthesis of secondary metabolites” mainly the “Cysteine and methionine metabolism” and the “Selenocompound metabolism” (Figure 5B,C). The other network formed a hub of eleven interacting proteins involved in “Protein processing in endoplasmic reticulum”, “Protein export”, “Carbon metabolism” and the “Peroxisome” pathways in 400 mM NaCl plants (Figure 5B). The network of proteins found in 600 mM NaCl plants clustered 19 proteins mainly involved in the same pathways (Figure 5C).

Analysis of PPI networks in NOD^+^ identified one main group of four interacting proteins in 200 mM NaCl plants, involved in “Metabolic pathways” and “Biosynthesis of secondary metabolites” mainly the “Cysteine and methionine metabolism” and the “Selenocompound metabolism” mentioned above (Figure 5D). The same network was identified with the enhancement of NaCl to 400 mM (Figure 5E) and 600 mM (Figure 5F) although a secondary network linked six proteins in 400 mM plants and eight proteins in 600 mM plants involved in “Protein processing in endoplasmic reticulum” and “Plant-pathogen interaction” pathways”.

## 3. Discussion

Salt stress in *Casuarina glauca* induces a complex array of physiological and biochemical responses that collectively lead to stress tolerance [6,13]. These include photosynthetic adjustments related to the functioning of the photosynthetic machinery [4], as well as the activity of antioxidant enzymes closely related to maintenance of cell membrane integrity [21], osmotic balance [7,8], and lipid matrix remodeling [21]. In order to complement these studies, in this work we have examined the effects of increasing salt concentrations (0, 200, 400 and 600 mM NaCl) on the protein profiles of branchlets from non-nodulated (KNO_3_^+^) and nodulated (NOD^+^) *C. glauca*.

Although the number of proteins might seem relatively low, it should be noted that the number of genes being expressed in each cell at a given time point in several systems only equals to ca. 50%. On top of this, the dynamic range is cell-dependent and protein extraction can be quite challenging. Our protein extraction protocol was optimized for consistency (low variability between technical replicates) in detriment of extracting more proteins, but with higher variability. Moreover, in proteomics studies the identification is mainly based on the stochastic selection from most abundant to least abundant ionized peptides. The results presented in this manuscript are based on identifications with an FDR of 5% according to [33,34,35,36,37], i.e., more stringent. Therefore, the quantification was based on even more stringent parameters, as only peptides with at least five transitions and an FDR of 1% are presented. In summary, in this study we have shown that the SWATH-MS methodology is suitable to plant proteomics, privileging the quality of the data instead of including higher number of proteins with lower confidence.

Among the 357 quantified proteins, 43 were regulated by salt stress in KNO_3_^+^ plants and 25 in NOD^+^ plants, 19 of which were common to both groups (Figure 1a,b). The first important information regards the stability of most of the identified proteins, likely linked to the maintenance of plant performance under salt stress [6,12,13,21]. Additionally, such a low number of differentially expressed proteins (DEPs) might be explained by the assumption that *C. glauca* has constitutive defense mechanisms against various stresses and that defense responses are activated during the early stages of stress imposition [21]. Moreover, the difference between KNO_3_^+^ and NOD^+^ plants regarding the number of DEPs might be associated with the fact that the process of nodulation in *C. glauca* leads to a set of defense-related events, such as the transcriptional activation of defense-related genes encoding proteins involved in ROS-detoxification [2,3], or the protection of nodules against pathogenic microorganisms [3,38], probably triggering a systemic defense response. On the other hand, the fact that at 200 mM NaCl *Frankia* was no longer fixing nitrogen in *C. glauca* nodules, and thus at this salt concentration the plants were subjected to both salt and mineral stresses [12], may explain the slightly higher induction of DEPs in NOD^+^ than in KNO_3_^+^ at 200 (24% vs. 16%) and 400 mM (56% vs. 44%), which was then similar at 600 mM NaCl (93%) when major physiological changes related to photosynthesis, membrane stability and osmoprotection mechanisms are induced [4,7,8,13,21,22]. At any rate, in both plant groups, the number of DEPs was regulated in a gradual manner, accompanying the severity increase of salt stress imposition, likely reflecting changes towards acclimation to high salt exposure.

In both KNO_3_^+^ and NOD^+^ groups, most of the DEPs were multifunctional and the dominant categories included metabolic and cellular processes (Biological Processes), catalytic and binding proteins (Molecular Functions), and cell and organelle proteins (Cellular Components) (Figure 2). These results are in line with those observed in woody Mediterranean species exposed to drought and salinity [39]. Thus, it would be interesting to understand whether independently of the species endemism (tropical or Mediterranean), the mechanisms underlying the response of woody plants to abiotic stress follow similar biochemical pathways.

In general, the functional categories of DEPs were similar in both plant groups with major differences regarding carbohydrate (nearly doubled in KNO_3_^+^ compared to NOD^+^) and energy (not found in NOD^+^) metabolism. According to Duro et al. [12], in NOD^+^ plants nitrogen-limiting conditions are imposed already at 200 mM, since at this NaCl concentration *Frankia* nitrogen fixation activity is reduced to residual levels. These conditions lead to a double-stress condition (salinity and N-deficiency), imposing a concomitant stronger negative effect on the expression of genes involved in carbohydrate metabolism [12], photosynthesis [4], as well as to an accumulation of sugars [7] at this salt concentration. Nevertheless, at higher salt concentrations (400 and 600 mM), NOD^+^ plants seemed to be able to acclimate through osmotic adjustments (to maintain a potential gradient of water influx and sustain metabolic activity) [4,7], facilitating cellular homeostasis, detoxification and survival under stress [15]. Based on these data, and considering that the levels of most proteins involved in carbohydrate metabolism remained constant in NOD^+^ plants along the stress imposition, it is likely that the nodulated plants reduced their metabolic activity earlier than the non-nodulated plants, thus minimizing the expenditure of energy. This is consistent with the observation that, in opposition to NOD^+^ plants, in the KNO_3_^+^ series, a strong upregulation of several enzymes was observed. Among them were those involved in sugar degradation (glycolytic enzymes), like glyceraldehyde-3-phosphate dehydrogenase, triosephosphate isomerase and enolase, as well as citric acid cycle related enzymes, like malate dehydrogenase and malic enzyme, and also a mitochondrial ATP synthase subunit. Similarly, two photosynthesis-related proteins, a ribulose-1,5-bisphosphate carboxylase/oxygenase (RuBisCO) chaperone and a 19 kDa protein from the thylakoid lumen with unknown function (and weak homology with PsbP), were upregulated under increasing salt stress in the KNO_3_^+^ group, but not in the NOD^+^ group. It should however be highlighted that in both KNO_3_^+^ and NOD^+^ plants, ca. 80% of the set of 357 proteins were constitutively expressed and mainly represented by proteins involved in carbon metabolism, including photosynthesis, as well as stress-related proteins, including antioxidant enzymes (Table S4). This might reflect the ability of *C. glauca* to cope with adverse environmental conditions suggesting that many defense mechanisms are constitutively active in this species.

The hierarchical clustering of DEPs showed an obvious enrichment of proteins involved in the metabolic pathways cited above (Figure 4). In agreement, the networks found in protein–protein interaction analysis were mainly involved in general metabolic pathways and biosynthesis of secondary metabolites (Figure 5). The increase in the number of proteins ultimately accumulating at 600 mM NaCl, tended to start at lower NaCl levels in NOD^+^ than in KNO_3_^+^ plants (Figure 4C). It is plausible to postulate that in nodulated plants, osmotic stress defense mechanisms are induced earlier than in control plants. Firstly, nodulated plants activate anti-pathogen defense mechanisms when they develop nodules, which are likely to include systemic responses [2,3,37,40,41,42]. Secondly, since *Frankia* nitrogenase activity is reduced dramatically from 200 mM NaCl upwards [12,13], and thus the nodulated plants are exposed to N-deficiency at this point, not only to salinity stress. Additionally, in plants growing on potassium nitrate, the translocation of sodium ions from the root to the shoot might be impaired due to an effect of nitrate on shoot potassium homeostasis [43].

The adaptation to biotic and abiotic stress requires the integration of redox- and reactive oxygen species (ROS) signaling and metabolic activities. Previous results on antioxidant defense had shown that the non-enzymatic defense system could not explain the apparent salt tolerance of *C. glauca*, as ascorbate levels in branchlets declined with increasing salt imposition in both KNO_3_^+^ and NOD^+^ plants [21]. On the other hand, several antioxidant enzyme activities were strongly increased in both groups from 200 mM NaCl onwards [21]. This is in line with the observations from this study, where several enzymes involved in antioxidant defense (two different ascorbate peroxidases, monodehydroascorbate reductase, superoxide dismutase) started to accumulate in branchlets of KNO_3_^+^ plants at 400 mM NaCl, whereas in NOD^+^ plants only monodehydroascorbate reductase levels increased (starting at 200 mM NaCl). Besides that, in both groups the levels of peroxiredoxins increased at the two highest salt concentrations while the relative amount of a set of anti-oxidant enzymes remained quite stable along the stress imposition. Thus, despite the fact that catalase levels were reduced consistently under salt stress in branchlets of both KNO_3_^+^ and NOD^+^ plants, the capacity for ROS detoxification seemed to be in place, as also demonstrated by the membrane stability and photosynthetic potential maintenance [4,21]. Consistent with that, a 14-3-3 protein and a nucleoside diphosphate kinase (NDPK) were upregulated in both plant groups. These proteins have been implicated in abiotic stress response, e.g., in hydroperoxide-responsive signaling pathways in leaves [44,45]. This might imply that this pathway is active in salt-stressed *C. glauca* plants independent of whether the plants are N-deprived or not and may explain the reduction of catalase levels which would be required for the increased production of hydrogen peroxide for signaling.

Since antioxidant defense involves the accumulation of two important thiol-containing compounds, cysteine and glutathione [46,47], we can expect sulfur metabolism to change under salt stress imposition. In this context, it is interesting to note that recent studies suggested the existence of a dynamically interacting module in the chloroplast stroma consisting of a peptidylprolyl isomerase (cyclophilin), two 2-cysteine peroxiredoxins A/B and a cysteine synthase, that integrate sulfur metabolism and oxylipin signaling during the acclimation to high light stress [48]. In salt-stressed KNO_3_^+^ plants, we also found the upregulation of two 2-cysteine peroxiredoxins, a peptidylprolyl isomerase, and a cysteine synthase, indicating that this module is also active in the salt stress response of *C. glauca.* The parallel increase of the levels of allene oxide cyclase (AOC), an enzyme of the oxylipin pathway that leads to the synthesis of jasmonic acid and its precursor 12-oxo phytodienoic acid (*OPDA*) [49], supports this hypothesis. Interestingly, a chloroplast envelope quinone-oxidoreductase homolog (ceQORH) was induced during salt imposition in both the KNO_3_^+^ and the NOD^+^ series. The Arabidopsis homolog of this enzyme has been implicated in the reduction of long-chain γ-ketols spontaneously produced in the chloroplast from the unstable allene oxide formed by the enzyme preceding AOC in the oxylipin pathway, allene oxide synthase [50]. The upregulation of this ceQORH homolog indicates that in spite of the upregulation of AOC, its activity is limiting because otherwise long-chain γ-ketols could not accumulate.

With regard to enzymes involved in sulfur metabolism, the apparent downregulation of methyltetrahydropteroyltriglutamate-homocysteine methyltransferase, also called B12-independent methionine synthase, involved in the regeneration of methionine from homocysteine, under salt stress in both KNO_3_^+^ and NOD^+^ groups is rather surprising since the upregulation of this particular enzyme has been linked to salt tolerance in Arabidopsis, cotton and rice [51,52,53]. Furthermore, a reduction in methionine synthesis under salt stress conditions would be expected to lead to the depletion of S-adenosyl-methionine which would not be consistent with the fact that polyamines accumulate in branchlets of *C. glauca* under these conditions [7]. So, this result might indicate that other members of the B12-independent methionine synthase protein family were upregulated under salt stress but were not detected in this study or that this function is undertaken by one of the multifunctional DEPs.

Interestingly, some universal stress-related proteins were down-regulated with increasing salt stress imposition, e.g., heat shock protein 80 in the NOD^+^ series and heat shock protein 90 (endoplasmin) in both series. Also, the mitochondrial NAD^+^-dependent formate dehydrogenase, linked to plant stress responses [54], was only induced with increasing salt stress in the KNO_3_^+^ series. However, salt stress led to upregulation of PHOS32 and PHOS34 in both plant groups, and of another homolog of bacterial universal stress response protein A in the NOD^+^ group. A thaumatin, also linked to the response to various abiotic stresses [55], was induced in response to salt stress in both series, while a homolog of temperature-induced lipocalins, which are also linked to the abiotic stress response [56], was upregulated only in the NOD^+^ group. A glycine-rich RNA binding protein, member of a protein family involved in various stress responses [57], was only upregulated in the KNO_3_^+^ series while an annexin, a member of a family of calcium- and phospholipid-binding proteins that has been linked to various stress responses [58], was upregulated in both groups. A protein disulfide isomerase, which is involved in the endoplasmic reticulum stress response that is also linked to salt stress [59,60], was only upregulated under salt stress in the KNO_3_^+^ group. In summary, while some stress response pathways seem to be active in both series, others were only active either in non-nodulated plants growing on nitrate as N source, or in nodulated plants.

Cytoskeleton proteins are also important components of plant stress response [61,62]. Despite the fact that a tubulin alpha chain was downregulated in both series along the stress imposition, the level of five other tubulins remained stable (Table S4). In combination with the electrolyte leakage analysis [21], our results suggest that this set of proteins is associated with cell integrity through microtubule stabilization [62]. Similarly, in the case of cell wall proteins, despite the fact that one β-1,3-glucosidase is down-regulated in both plant groups, the levels of other related proteins, like β-1,3-glucanases, remained unchanged suggesting a role of these proteins in cell wall remodeling imposed by high salt concentrations [63,64].

Overall, the mechanisms of salt-tolerance in *C. glauca* are nearly similar to those observed Azri et al. [65] in the facultative halophyte *Aeluropus littoralis* (Poaceae), and by Qiao et al. [66] in the salt-tolerant Chinese Willow (*Salix matsudana*). Both systems also showed adjustments in the photosynthetic machinery, C/N metabolism and energy-producing processes, together with the reinforcement of the antioxidant mechanisms to maintain membrane functioning and cellular homeostasis. However, it should be highlighted that this comparison only refers to the photosynthetic organs and that according to Qiao et al. [66] the proteome of *S. matsudana* roots is more responsive to salt than that of leaves, particularly regarding the biosynthesis of secondary metabolites. In line with this observation, we have previously identified several primary metabolites in roots of *C. glauca* (subjected to the same salt conditions of the present study) with a probable role in osmoprotection and nutrient transport [7]. Additionally, an integrative analysis (physiology, biochemistry, metabolomics, proteomics and transcriptomics) of the impact of salt stress in *C. glauca* branchlets will be concluded in the near future. This will particularly be focused on the photosynthetic machinery adjustments and membrane stability and dynamics, which are among the first and major stress targets and are involved in the process of stress perception by the plants [67]. Furthermore, running efforts will extend the throughout analysis to roots and nodules of *C. glauca*. Since the plant growth system used (hydroponic culture) does not mimic the natural environmental conditions, we think that while the data produced thus far constitute a strong baseline to understand the mechanisms used by *C. glauca* to cope with salt stress, further studies should be performed under conditions closer to those found in natural ecosystems (e.g., soil types, temperature, water) and include the analysis of the interaction between different environmental stresses, plants ecotypes, and *Frankia* strains. In particular, the identification of a *Frankia* strain that shows high salt tolerance in planta is required. Ngom et al. [9] have shown that the salt tolerance of *Frankia* strains *in vitro* does not predict their salt tolerance *in planta*; however, they used a *C. glauca* ecotype with lower salt resistance than the one used in our lab [4,12,21]. Future experiments should include plant and bacterial ecotypes/strains with different levels of salt resistance.

## 4. Materials and Methods

### 4.1. Growing Conditions and Salt Treatment of Casuarina glauca

*C. glauca* clonal plants were grown in Broughton and Dillworth’s (BD) medium under the conditions previously described [4]. For the implementation of salt-stress, symbiotic (nodulated by *Frankia* strain Thr [68]) (NOD^+^) and non-symbiotic (supplemented with mineral nitrogen) (KNO_3_^+^) six month-old plants were transferred to a walk-in growth chamber (10000 EHHF, ARALAB, Portugal) under environmental controlled conditions of temperature (26/22 °C), photoperiod (12 h), relative humidity (70%), external CO_2_ levels (380 µL L^−1^) and irradiance (ca. 500 µmol m^−2^ s^−1^). Plants not supplemented with NaCl were maintained as controls. To avoid osmotic shock, enhancement of salt levels was gradually imposed through the addition of 50 mM NaCl per week to the nutrient solution until concentrations of 200, 400 and 600 mM were obtained. The hydroponic nutrient solutions were renewed twice per week in order to balance the nutrient and salt concentrations as well as the pH. The plants were maintained for one week on each NaCl concentration. To ensure that the plants had the same age at the time of analysis, the 600 mM group of plants was the first to be gradually treated with NaCl; when this group surpassed 200 mM NaCl, salt stress implementation was initiated in the 400 mM group and so forth. All procedures have been described before [4]. For the proteomic analysis, branchlets from four plants per treatment were harvested, frozen immediately in liquid nitrogen and stored at −80 °C before protein extraction.

### 4.2. Protein Extraction and Quantification

Proteins from branchlets were extracted and quantified using the methods reported before [69] with slight modifications. Briefly, 1.0 g of plant frozen branchlets was finely ground in liquid nitrogen using a mortar and pestle and mixed with 7% (*w*/*w*) polyvinylpolypyrrolidone. The fine powder was homogenized in 10 mL of 10% trichloroacetic acid (TCA), 0.07% 2-mercaptoethanol (2-ME) in cold acetone, and sonicated on ice (3 pulses of 5 s each, 70% output, 10 s intervals) using an Ultrasonic processor (Gex 130, 130 W, Vernon Hills, IL, USA). This process was repeated four times at intervals of 10 min. The mixture was incubated overnight at −20 °C for complete precipitation of proteins and then centrifuged (10,000× *g*, 4 °C, 10 min). The supernatant was discarded, and the pellet was washed three times with TCA/2-ME. The pellet was completely re-suspended in the same solution and centrifuged (10,000× *g*, 4 °C, 10 min). The final pellet was dried at room temperature and resuspended in 10 mL of extraction buffer of 30% sucrose, 2% sodium dodecylsulfate (SDS), 0.1 M TRIS-HCl, pH 8.0, 5% 2-ME). After the addition of 5 mL of phenol saturated with 0.1 M TRIS-HCl pH 8.0, the samples were centrifuged (10,000× *g*, 4 °C, 15 min), and the obtained supernatant was transferred to a new tube and stored on ice. The proteins from the lower phase were extracted twice with one volume of a 2:1 mixture of extraction buffer and phenol. The phenol phases from the three extractions were grouped and incubated overnight at −20 °C with five volumes of 0.1 M ammonium acetate in methanol. Total proteins were pelleted by centrifugation (12,000× *g*, 4 °C, 20 min), washed twice with 1 M ammonium acetate in methanol, twice with acetone, once with 80% ethanol, and dried at room temperature. In each washing step, the samples were centrifuged (10,000× *g*, 4 °C, 5 min). The remaining pellets were dissolved in solubilization buffer [7 M urea, 2 M thiourea, 60 mM dithiothreitol (DTT), 4% (*w*/*v*) CHAPS (3-[(3-Cholamidopropyl)dimethylammonio]-1-propanesulfonate)] using 1 mL of buffer per 50 mg pellet, at room temperature for 2 h under shaking, and then centrifuged (20,000× *g*, 4 °C, 30 min). Proteins were quantified using the 2-D Quant Kit according to the manufacturer’s instructions (GE Healthcare, Alfragide, Portugal).

### 4.3. Sample Preparation for Mass Spectrometry (MS) Analysis

Denatured samples were alkylated with acrylamide and subjected to in-gel digestion following the short-GeLC approach [30]. Briefly, samples were loaded onto a “4–20% TGX Stain-Free Gel” (Bio-Rad, Amadora, Portugal), followed by partial electrophoretic separation (SDS-PAGE). Proteins were subsequently visualized with Colloidal Coomassie Blue staining [70]. Gel lanes were sliced into seven bands of equal size and further sliced into small pieces for independent processing. Gel pieces were destained, dehydrated, and rehydrated with 75 μL of trypsin solution (0.01 μg μL^−1^ trypsin in 10 mM of ammonium bicarbonate). Protein digestion was performed overnight at room temperature, and digested peptides were extracted from the gel by sequential incubation with acetonitrile (ACN) solutions (30%, 50%, and 98%) in 1% formic acid (FA). Peptides extracted from different bands were pooled together in two peptide mixtures per sample for subsequent liquid chromatography (LC)-MS/MS analysis. Peptide mixtures were dried and desalted using OMIX tips with a C18 stationary phase (Agilent Technologies, Lisbon, Portugal). To monitor losses during sample preparation, samples were spiked with 1 μg of recombinant green fluorescent protein (GFP) before digestion.

### 4.4. Protein Sequential Window Acquisition of All Theoretical Mass Spectra (SWATH-MS)

#### 4.4.1. SWATH Acquisition

Samples were analyzed on a Triple TOF^TM^ 5600 System (Sciex^®^, Singapore) through information-dependent acquisition (IDA) followed by SWATH-MS. Peptides were resolved by LC (nanoLC Ultra 2D, Eksigent^®^, Livermore, CA, USA) on a ChromXP^™^ C18AR reverse phase column (300 μm ID × 15 cm length, 3 μm particles, 120 Å pore size, Eksigent^®^) at 5 μL min^−1^, and eluted into the mass spectrometer with an ACN linear gradient in 0.1% FA (2% to 30% ACN, for 45 min) using an electrospray ionization source (DuoSpray^™^ Source, Sciex^®^, Singapore). Pooled mixtures were analyzed in IDA mode to generate peptide fragmentation spectra for further protein identification/library creation. For IDA, the mass spectrometer was set to scanning full spectra (350–1250 m/z) for 250 ms, followed by up to 20 MS/MS scans (100–1500 m/z). Candidate ions with a charge state between +2 and +5, and counts per second above a minimum threshold of 70, were isolated for fragmentation. One MS/MS spectrum was collected for 100 ms before adding those precursor ions to the exclusion list for 15 s (mass spectrometer operated by Analyst^®^ TF 1.7, Sciex^®^, Singapore). The rolling collision energy was used with a collision energy spread of 5.

For quantitative analysis, the peptide mixtures were combined into a single sample per biological replicate. The SWATH-MS setup was essentially as described by [31]. The mass spectrometer was operated in the looped product ion mode and specifically tuned to allow a quadrupole resolution of 25 m/z mass selection. Using an isolation width of 26 m/z (containing 1 m/z for the window overlap), a set of 30 overlapping windows was constructed, covering the precursor mass range of 350–1100 m/z. A 100 ms survey scan (350–1250 m/z) was acquired at the beginning of each cycle, and SWATH-MS/MS spectra were collected from 100–1500 m/z for 100 ms resulting in a cycle time of 3.1 s. The collision energy for each window was determined according to the calculation for a charge +2 ion-centered upon the window with a collision energy spread of 15.

#### 4.4.2. Protein Identification/Library Generation

Peptide identification and library generation were performed with Protein Pilot software (v5.0, Sciex^®^, Singapore), against the transcriptome database of *C. glauca* (https://www.ncbi.nlm.nih.gov/bioproject/397052).

An independent False Discovery Rate (FDR) analysis, using the target-decoy approach provided by Protein Pilot^TM^, was used to assess the quality of identifications. Positive identifications were considered when identified proteins and peptides reached a 5% local FDR [33,34,35,36,37]. A specific library of precursor masses and fragment ions was created by combining all files from the IDA experiments, and used for subsequent SWATH-MS processing. Data processing was performed using SWATH^TM^ processing plug-in or PeakView^TM^ (v2.0, Sciex^®^, Singapore).

### 4.5. Statistical Analysis

Statistical analysis was performed in IBM SPSS software (v22). The Mann-Whitney test was applied for comparisons between experimental groups and statistical significance was considered for p-values below 0.05 and a fold change of 1.5 (0.67 > ratio > 1.5).

### 4.6. Functional Annotation and Classification of Identified Proteins

To determine the functional classification and biological properties of identified DEP, the sequences were mapped to gene ontology (GO) terms. For this, a homology search was performed for all the identified sequences with a localized NCBI BLAST searched against the NCBI nr protein database for homologous sequences using a query-friendly version of blast (QBlast) (http://www.ncbi.nlm.nih.gov/protein). The proteins identified were displayed referring to the homologs with the highest sequence similarities. GO annotation was performed using the online bioinformatics platform BLAST2GO (https://www.blast2go.com/) [71,72]. The identified proteins were classified into three functional categories: CC (cellular components), BP (biological processes) and MF (molecular functions). In addition, all mapped sequences were annotated using the KEGG (Kyoto Encyclopedia of Genes and Genomes) database accessions to obtain protein domain information.

### 4.7. Hierarchical Cluster Analysis of Protein Abundance and Interaction Network Analysis

Protein abundance values were estimated using PermutMatrix (v. 1.9.2) software [73] where dissimilarity was measured using Euclidian distance, and the hierarchical clustering was performed using the average linkage rules with data normalized to rows. Three comparisons were made along the salt stress exposure within (i) non-nodulated (KNO_3_^+^), (ii) nodulated (NOD^+^); and (iii) KNO_3_^+^ vs. NOD^+^ plants at each salt concentration. Protein-protein interaction (PPI) networks of DEP were constructed using the String database (version 11.0) (http://www.string-db.org/) with confidence scores higher than 0.7. Genes that did not interact with any others were removed.

## 5. Conclusions

In conclusion, our study is a step forward in the understanding of the mechanisms underlying the robust salt stress tolerance in *Casuarina glauca*. The first major finding was related with the minimal impact of heavy salinity in the proteome of branchlets, as disclosed by the stable expression of the vast majority of the quantified proteins, i.e., 289 out of 357, and by the fact that multifunctional key proteins, and associated networks, were particularly regulated at the highest stress levels (400 and 600 mM), towards acclimation to salt stress. Secondly, the main differences found between non-nodulated (KNO_3_^+^) and nodulated (NOD^+^) plants are most likely associated with an earlier induction of a set of defense-related events in the latter group, i.e., during the nodulation process and after the symbiosis in turned to residual levels (at 200 mM NaCl). Overall, the results are in line with previous studies from our group, confirming that the tolerance of *C. glauca* to salt concentrations above sea level is related to the maintenance of proteome stability and the triggering of antioxidative defense mechanisms. This allowed plants to maintain a high physiological and biochemical performance, particularly as regards the photosynthetic machinery that in turn promoted cellular metabolism and homeostasis.

## Supplementary Materials

Supplementary materials can be found at https://www.mdpi.com/1422-0067/21/1/78/s1.

## Abbreviations

DEPs	Differentially Expressed Proteins
GO	Gene Ontology
KEGG	Kyoto Encyclopedia of Genes and Genomes
KNO_3_^+^	Non-nodulated *Casuarina glauca* supplied with KNO^3^
NOD^+^	*C. glauca* nodulated by nitrogen-fixing *Frankia* Thr
Short-GeLC-MS/MS	Short Gel, Long Gradient Liquid Chromatography Tandem Mass Spectrometry
SWATH-MS	Sequential Window Acquisition of all Theoretical Mass Spectra
